# Reversible Ultrathin PtO_*x*_ Formation at the Buried Pt/YSZ(111) Interface Studied In Situ under Electrochemical Polarization

**DOI:** 10.1021/acs.jpclett.2c03614

**Published:** 2023-02-17

**Authors:** Vedran Vonk, Sergey Volkov, Thomas F. Keller, Alexander Hutterer, Pirmin Lakner, Florian Bertram, Jürgen Fleig, Alexander K. Opitz, Andreas Stierle

**Affiliations:** † Centre for X-ray and Nanoscience CXNS, Deutsches Elektronen-Synchrotron DESY, Notkestr. 85, 22607 Hamburg, Germany; ‡ Deutsches Elektronen-Synchrotron DESY, Notkestr. 85, 22607 Hamburg, Germany; § Institute of Chemical Technologies and Analytics, Technische Universität Wien, 1060 Vienna, Austria; ∥ Physics Department, University of Hamburg,, 20355 Hamburg, Germany

## Abstract

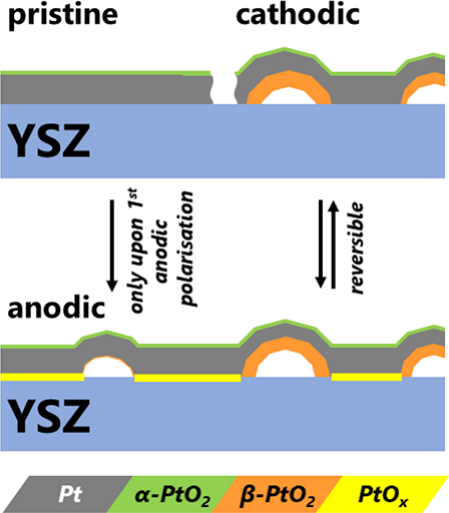

Three different platinum oxides are observed by in situ X-ray diffraction during electrochemical potential cycles of platinum thin film model electrodes on yttria-stabilized zirconia (YSZ) at a temperature of 702 K in air. Scanning electron microscopy and atomic force microscopy performed before and after the in situ electrochemical X-ray experiments indicate that approximately 20% of the platinum electrode has locally delaminated from the substrate by forming pyramidlike blisters. The oxides and their locations are identified as (1) an ultrathin PtO_*x*_ at the buried Pt/YSZ interface, which forms reversibly upon anodic polarization; (2) polycrystalline β-PtO_2_, which forms irreversibly upon anodic polarization on the inside of the blisters; and (3) an ultrathin α-PtO_2_ at the Pt/air interface, which forms by thermal oxidation and which does not depend on the electrochemical polarization. Thermodynamic and kinetic aspects are discussed to explain the coexistence of multiple phases at the same electrochemical conditions.

Considered a noble metal with significant stability and notable catalytic activity, platinum is an attractive electrode material in various electrochemical applications. In particular for electrocatalysis, which has become ever more important in view of a sustainably green economy, platinum-based electrodes play an important role. In such applications, the electrodes typically undergo redox cycles and the long-term stability is problematic.^1^ It is discussed that platinum oxide formation is involved in various mass transport phenomena relevant for electrode failure,^2^ which calls for detailed mechanistic studies of the oxidation process. Although platinum is relatively stable under thermal oxidation conditions,^3,4^ its use under more harsh electrochemical conditions can lead to severe degradation,^2,5^ such as loss of mechanical stability and electrode delamination from its solid support. It has also been observed that the platinum oxidation process can be drastically different when comparing bulk platinum electrode surfaces with nanoparticles or buried electrode interfaces.^6,7^ Most likely, some of the notoriously high kinetic barriers^8^ are lowered substantially in reduced dimensions or at buried interfaces. The initial oxidation of free platinum surfaces has been observed to proceed by the formation of an ultrathin oxide in the case of thermal oxidation at elevated pressure^9^ and under electrochemical conditions.^10^ Within a particular thermodynamic (electrochemical) range, the formation of this oxide layer is reversible and the so-called place–exchange mechanism^11,12^ can be used to understand this phenomenon. For slightly higher potentials, the process enters a regime of irreversible 2D oxide formation, the details of which, such as structure and phase, are not yet fully uncovered, before at even higher potentials bulk Pt-oxide starts to form.^13^

The aformentioned issues also seem to play a role for the oxidation and stability of Pt at the buried interface with yttria-stabilized zirconia (YSZ). Such electrode interfaces occur in the field of high-temperature electrocatalysis where they are frequently encountered in solid oxide cells such as oxygen sensors commonly called Lambda probes. In contrast to free platinum surfaces, mechanistic studies of the oxidation process at the Pt/YSZ interface have been difficult because it represents a deeply buried interface.

Using cyclic voltammetry (CV) on Pt/YSZ electrodes, different features have been observed, emerging as soon as the sample is subjected to an initial anodic polarization step.^14,15^ Depending on the details of the polarization, such as extent of the applied bias voltage and duration of the polarization, different irreversible and reversible oxidation processes have been discussed.^14^ Moreover, it has been reported that the suspected formation of new interfacial oxide species caused a change of electrochemical impedance spectra recorded on Pt/YSZ electrodes.^16,17^ But nevertheless this still needs to be regarded as an indirect proof only and a direct experimental observation of the presence of an ultrathin oxide at the buried Pt/YSZ interface has hitherto not been reported. Therefore, demonstrating the existence of platinum oxides at the interface of Pt and YSZ and their effect on the oxygen exchange kinetics of this electrode material would represent an important step in understanding this electrode system.

Here, we present in situ X-ray reflectivity (XRR) and grazing incidence X-ray diffraction (GIXRD) experiments utilizing synchrotron radiation and a special setup, which allows for heating and controlling the electrochemical conditions. The results show that an approximately 1 nm thin platinum oxide layer forms at the buried Pt/YSZ interface upon applying an anodic polarization of +180 mV. The XRR data indicate that the formation and dissolution of this ultrathin oxide at the buried interface is reversible while cycling the electrochemical potential. By combining the results of XRR and GIXRD, the latter of which shows that the outer Pt electrode surface also oxidizes, it is possible to clearly disentangle the formation of oxides at the two interfaces.

Platinum films were deposited by magnetron sputtering (BAL-TEC MED 020; Pt target: 99.95% pure) onto YSZ(111) substrates (miscut angle < 0.1°, Crystec, Berlin) in 2 × 10^–2^ mbar Ar atmosphere at elevated temperature. Heating was done by a boron nitride heater (Boralectric HT-1001) operated at 1223 K set temperature resulting in ca. 1023–1073 K substrate temperature. The growth conditions were chosen such that epitaxial single crystal thin films were achieved, following known recipes.^18^ Typical XRD measurements performed in the laboratory while optimizing the growth can be found in the Supporting Information. This has the advantage that the buried interface is smooth and well-defined, because over the whole sample area it will consist of (111)-oriented Pt in contact with YSZ(111), which is not the case for polycrystalline or textured metal films, where different orientations will coexist. In addition, the diffraction pattern of single crystal epitaxial electrodes reveals more structural details compared to the polycrystalline case. In order to obtain closed and gastight electrodes still accessible by X-rays, it is necessary to grow film thicknesses of the order of 100 nm.

For performing in situ X-ray experiments under electrochemical conditions, a dedicated sample chamber was constructed. It contains a custom-made resistive heater, allowing a maximum temperature of approximately 1000 K in controlled gas environment or in air. The chamber walls are made of Kapton foil, and it can be flushed with gases. Electrical feedthroughs allow for electrically contacting the sample. The free Pt surface is contacted by a needle, which can be moved with piezomotors inside the chamber. The counter electrode is contacted by clamping a wire between it and the heater and adding a small amount of additional Ag paste as an adhesive. A commercial potentiostat was used for controlled DC polarization, electrochemical impedance spectroscopy (EIS), and cyclic voltametry (CV) measurements. As, according to the impedance data, the polarization resistance of the thin film working electrode is orders of magnitude larger than the electrolyte and the counter electrode resistance, the resulting overpotential almost exactly amounts to the applied bias voltage. More information about this feature of treating the overpotential is given in refs (19−21). Moreover, the EIS results were used for retrieving the exact sample temperature, which was calculated from the measured ionic conductivity of the electrolyte using the approach described in ref (19). With this method, the sample temperature of all in situ experiments presented throughout this work was determined to be 702 ± 5K (note that the error bar is the result of an EIS fitting procedure).

All X-ray experiments were conducted at beamline P08^22^ of the PETRA III synchrotron facility (DESY, Hamburg, Germany). The X-ray energy was 25.000 keV (wavelength λ = 0.4972 Å), and it was focused to a size of 1 × 0.1 mm^2^ (HxV) on the sample. The chamber was mounted onto a 6-circle diffractometer, which allows for measurements in reciprocal space coordinates. Typical X-ray data consist of X-ray reflectivity (XRR) measurements and reciprocal space maps (RSMs). The latter measurements were done in grazing incidence geometry, which is beneficial for the signal-to-noise ratio and which is used to probe either the topmost 10 nm of the Pt electrode or its whole volume, thereby including the deeply buried interface with YSZ. From the XRR measurements, one can fit the in-plane-averaged electron density profile along the surface normal direction. From the off-specular RSMs, the different crystal lattices (lattice parameters and corresponding phases) and their relative orientation and nature (layers vs powderlike) are extracted.

Figure 1 shows X-ray reflectivity data taken at overpotentials of −500 mV and +180 mV. The curves show a fast oscillation, which are the so-called finite thickness fringes and which are due to interference of X-rays scattered at the top and bottom of the Pt thin film electrode. The period of these fringes is inversely proportional to the film thickness and does not change noticeably for the two polarization states. The most prominent difference between the two measurements is that for the data taken at an anodic overpotential of +180 mV (oxidizing conditions) an additional oscillation appears, modulating the overall shape with a relatively long period. This feature is a direct evidence of the formation of a new, relatively thin layer, with a density different from pure Pt. During several potential cycles, XRR measurements in an angular range close to the first maximum of this modulation (see Figure 1b) show that its appearance is fully reversible and that even the finite thickness fringes of the whole Pt electrode are recovered. Due to the large number of points that need to be taken to resolve the fringes, these measurements took about 1.5 h and as such represent static polarization conditions. Figure 1c shows the results of fitting electron density profiles to the recorded XRR data, using the GenX program.^23^ As common within the recursive Parratt formalism for X-ray reflectivity,^24^ different slabs are defined, which make up the structure. Here, a model using 3 slabs is fitted to the data taken at a cathodic overpotential of −500 mV and 4 slabs are used for the +180 mV data. For each slab, the real and imaginary parts of its scattering length density, i.e., electron density and absorption, thickness and roughness are defined as fit parameters. The fits reproduce the positions of minima and maxima very well, just as the intensities near the maxima. The intensities in the minima are less well reproduced over the whole measured range, which in general points to a more complex interface morphology than modeled by simple Gaussian roughnesses in combination with (angle-dependent) fininite resolution effects, which are not taken into account. As discussed below, indeed the surface morphology is complex, due to a local delamination process. The resulting projected electron density profile, shown in Figure 1c) represents the main fit result. Here, it is seen that the most prominent difference appears close to the Pt/YSZ interface, whereby there is an electron density reduction under oxidizing conditions (+180 mV). The fitted values of the electron density in a 1–1.5 nm thin region at the buried interface change from a value very close to that of bulk Pt ρ_e_ = 5.17 e/Å^3^ to a value of approximately ρ_e_ = 2.8 e/Å^3^. Calculations of the error estimates on the fitted electron densities lead to values, within the used model, of the order of a few percent, which needs to be taken into account when interpreting the results. For example, the fitted local Pt density near the interface at −500 mV is about 3% higher than bulk Pt and this value is on the limit of being significant. Still, it cannot completely be ruled out that this might partly be the result of compressive strain, which for a lattice matched epitaxial Pt(111)/YSZ(111) interface, where 4 rows of Pt atoms fit on 3 unit cells YSZ, would be 1.5%. The nearly 50% density reduction at +180 mV is far beyond this error bar and matches very well with the electron density ρ_e_ = 2.72–2.95 *e*/Å^3^ expected for PtO_2_.^25,26^ Please note that the value of the electron density alone cannot be used to conclude which platinum oxide phase is formed; for further phase analysis additional experiments were conducted that are discussed in the following sections. Oxide formation at the buried interface leads to lattice expansion and pushes the rest of the 100 nm thick Pt electrode away from the YSZ. This is seen in the position of the free surface shifting by 0.5 nm outward in Figure 1c).

**Figure 1:**
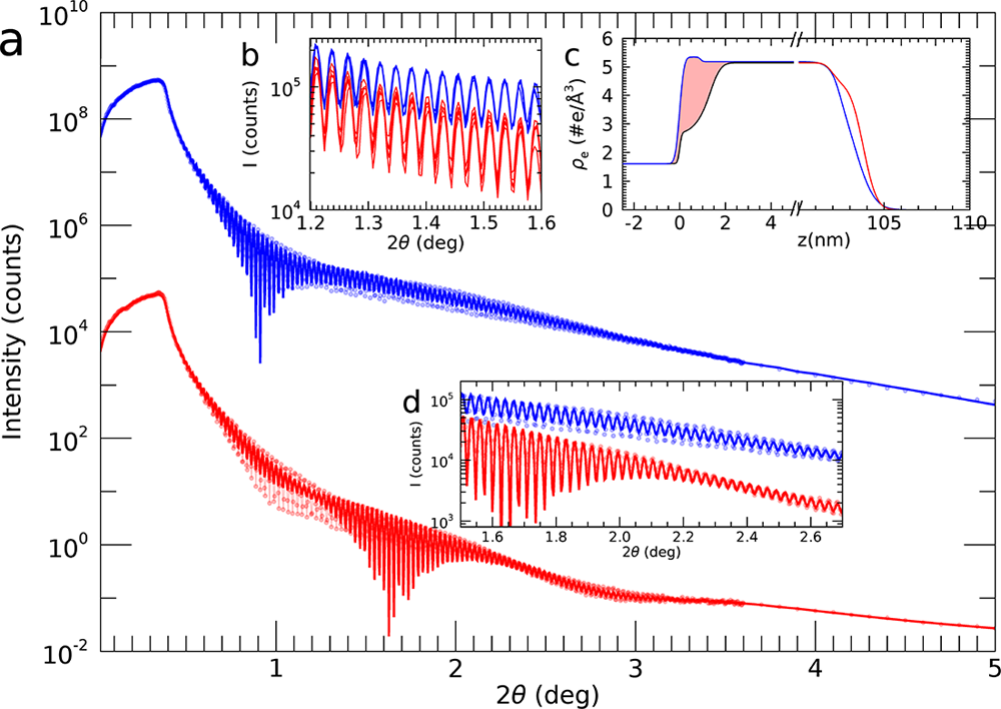
X-ray reflectivity curves and fit results. (a) Shown are the data (transparent connected points) and fits (thicker lines) taken at −500 mV (blue) and +180 mV (red) at an experimental sample temperature of 702 K (see text). The data taken at +180 mV are scaled by a factor of 10^–4^ for better clarity. (b) Part of the XRR curve for measurements taken at different times after several polarization cycles, highlighting the well-resolved fintite-thickness fringes, stability, and reversibility of the investigated interface. (c) Projected electron density profiles obtained from the fits at the two different polarization states. Note the broken *x*-axis, which is used to better highlight the two interfaces. Clearly, at the oxidizing conditions of +180 mV, the best fit result shows an electron density deficit at the interface (indicated by the shaded area), which is explained by the formation of a thin platinum-oxide layer. (d) Zoom of the data and fits, whereby the curves taken at −500 mV (blue) and +180 mV (red) are on a common scale.

The quasi-static polarization states discussed above showed that the ultrathin oxide formation at the buried interface is reversible. X-ray measurements during cyclic voltammetry, depicted in Figure 2, also indicates that the oxidation and reduction of the ultrathin oxide at the buried interface are fully reversible. The intensity at *Q* = 0.61 Å^–1^ on the XRR curve is measured during these potential sweeps. The *I*–*V* curce shows the anodic and cathodic peaks, which are characteristic for the formation and dissolution of a new species, which can now unequivocally be identified as an ultrathin platinum oxide layer at the buried interface. There is also excellent correlation between the scattered X-ray intensity and the recorded current, which both attain identical values when going through several cycles. The results from the XRR measurements and the corresponding analysis indicate that a thin platinum oxide layer forms at the buried interface upon anodic polarization. Further X-ray measurements show the presence of other platinum oxides, which, however, can be disentangled from that at the buried interface. These measurements can be performed such that either only scattering from the free Pt surface (Pt/air interface) is recorded or that the entire film is penetrated by the X-rays and (similar to the higher angle part of the XRR) both interfaces are illuminated. This is possible, because not only absorption but also refraction determines the penetration depth of X-rays into a medium.^27^ Here, when the X-ray beam makes an angle of 0.2° with the sample, the X-rays have a penetration depth Λ of only 10 nm into the Pt. When the angle of incidence is increased to 2.0°, the penetration depth increases to 520 nm, which is much larger than the total Pt film thickness. In this case, diffraction signals from YSZ are recorded, which also means that any scattering from the buried interface must be observable. Figure 3 shows the results of an in-plane reciprocal space map (RSM) taken at a grazing angle of 0.2°. In the Supporting Information, the RSM at 2.0° angle is shown together with more details. From all of the RSMs combined, it is concluded that the Pt electrode film has grown epitaxially but not pseudomorphically (the average Pt lattice parameters are completely relaxed), on the YSZ(111) substrate. The crystallographic directions are aligned as follows: [111]_Pt_∥[111]_YSZ_ along the surface normal and [11̅0]_Pt_∥[100]_YSZ_ in the in-plane direction. The different peaks in Figure 3 can be identified as belonging to metallic Pt and platinum oxide, which thus is present at the free electrode surface. In contrast to the oxide at the buried interface, which decomposes at the strongly reducing electrochemial conditions, the oxide at the surface is always observed both at anodic and cathodic polarizations. This indicates that the decay length of electrochemical activity from the triple phase boundary (TPB) is comparatively short. In the specific case, the decay of electrochemical activity from the TPB along the Pt surface is not associated with a laterally decaying electric field. Rather, it is connected with the surface diffusion of an adsorbed oxygen species on the Pt electrode. Hence, large parts of the Pt surface are obviously unaffected by the applied overpotential and its oxygen surface adsorbates are in quasi-equilibrium with the surrounding gas atmosphere.

**Figure 2:**
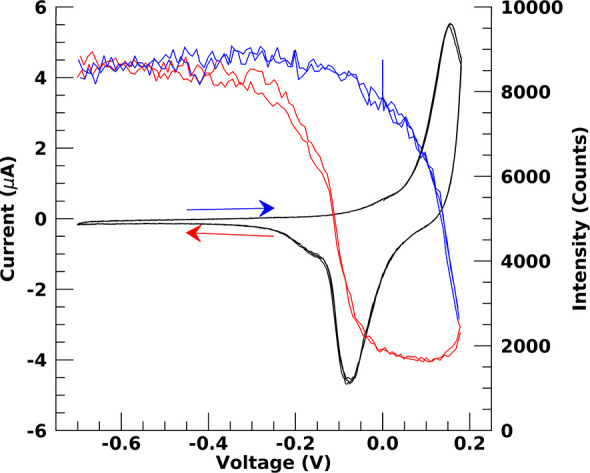
CV curves measured with a sweep rate of 5 mV/s during the in situ X-ray measurements and corresponing reflected intensity. Shown are the current vs voltage (black) and reflected X-ray intensity at a *Q* = 0.61 Å^–1^ (see Figure 1). Positive (blue) and negative (red) voltage scan directions are also indicated with the arrows. More details can be found in the main text and in the Supporting Information.

**Figure 3:**
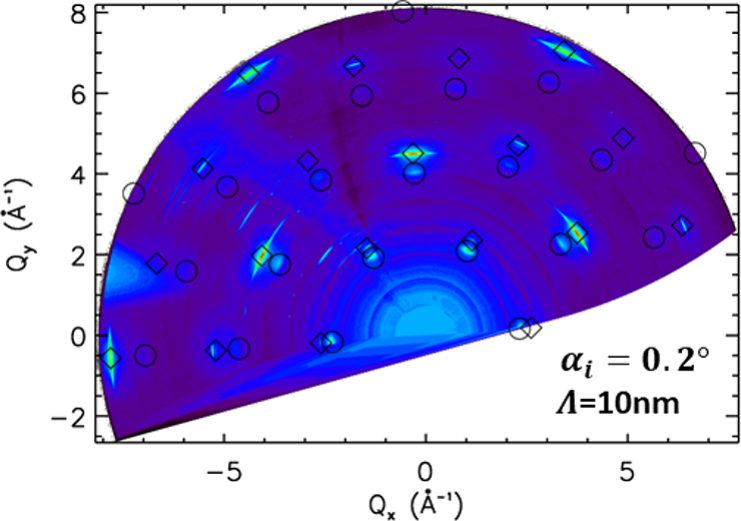
Reciprocal space map taken at 702 K and *U* = −500 mV polarization. At an incidence angle α_*i*_ = 0.2°, the X-ray beam penetrates only Λ = 10 nm into the Pt electrode, which leads to the observation of the CTR signal from the Pt metal (diamonds) and surface platinum oxide (circles). More information can be found in the Supporting Information.

Further structural details of the oxide at the surface are obtained from RSMs, which also include the out-of-plane direction (*Q*_*z*_); see Figure 4. Here, the plane including the Pt (0,1) crystal truncation rod (CTR) and the oxide is measured. The RSMs are plotted in the (*Q*_*r*_, *Q*_*z*_)-plane, whereby . The Pt CTR is clearly visible at *Q*_*r*_ = 2.6 Å^–1^ and is an indication that the Pt surface is very smooth. Along the (0,1) Pt CTR in Figure 4a, there is a weak Bragg peak at *Q*_*z*_ = 0.95 Å^–1^, which is attributed to part of the Pt film consisting of faults within the nominally ABC stacking along the (111) surface normal direction. From all RSMs, the angular positions of the different platinum peaks are used to determine the lattice parameter, resulting in *a*_Pt_ = 3.939(4) Å, which is in excellect agreement with the expected value based on the room temperature value *a*_0_ = 3.924 Å, thermal expansion α = 9.1 × 10^–6^ K^–1^, and temperature *T* = 702 K. At *Q*_*r*_ = 2.35 Å^–1^ the platinum oxide rod is seen as a streak along *Q*_*z*_. It appears that the direction of this rod is not parallel to the nearby Pt rods, both for the (0,1) and (1,0). Further inspection of the peak shape along *Q*_*r*_ at different positions *Q*_*z*_ shows that there are 2 overlapping diffraction signals. The fitted positions of these two signals show that one of them appears at a constant *Q*_*r*_ = 2.33 Å^–1^ and the other at a constant , which shows that the first originates from a 2D surface structure and the second from a bulk powderlike crystalline structure, which we both assume to belong to platinum oxide species. The in-plane *d*-spacings found from this procedure for the two oxides are *d*_ox1_ = 2.68(1) Å and *d*_ox2_ = 2.60(1) Å. Based on the symmetry of the in-plane diffraction pattern, which shows hexagonal symmetry and no domain formation, it is concluded that peaks of ox1 belongto α-PtO_2_. Further lattice parameter refinement including all of the in-plane peaks found in the RSM of Figure 3 leads to *a*_ox1_ = 3.105(4) Å. After a database search for the diffraction patterns of the different platinum oxides, PtO,^28−30^ PtO_2_,^25,26,30−34^ and Pt_3_O_4_,^32,35^ it is concluded that the value for *d*_ox2_ is closest to the nearly overlapping 101 and 011 reflections of β-PtO_2_,^34^ which crystallizes in space group *Pnnm* and which can be described by a distorted rutile-type structure. It needs to be mentioned that the value for *d*_ox1_ matches the reported value for α-PtO_2_ at room temperature. The value for *d*_ox2_ is larger than the reported ones for β-PtO_2_ and would match after taking into account a thermal expansion of (1–2) × 10^–5^ K^–1^. This finding might be explained by the difference in crystal structure; α-PtO_2_ is made up of hexagonal trilayers of PtO_2_, which are only weakly bonded along *z* and which are expected to have large ansisotropic thermal expansion coefficients. This feature, together with the epitaxial nature of the found α-PtO_2_ layer, might be responsible for neglible in-plane thermal expansion. The nearly rutile-type structure of β-PtO_2_, being much more isotropic in bond nature than α-PtO_2_, together with a bulklike polycrystalline apperance might explain that this structure does show measurable thermal expansion.

**Figure 4:**
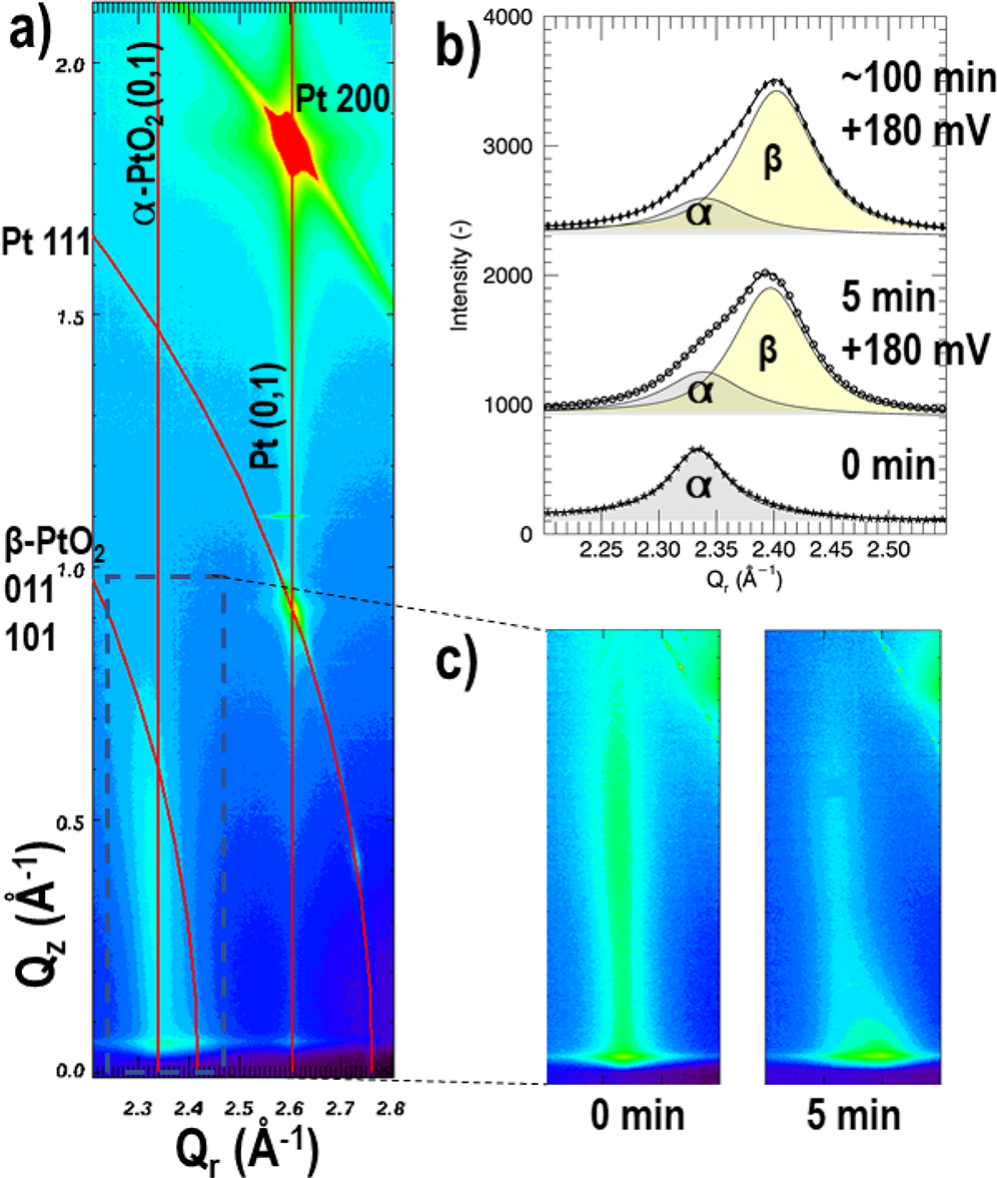
Reciprocal space maps taken at 702 K and different polarization times. (a) RSM showing the (0,1) Pt CTR at *Q*_*r*_ = 2.6 Å^–1^ and the nearby oxide rod at *Q*_*r*_ = 2.33 Å^–1^ after 5 min of anodic polarization at +180 mV. The indices of the different phases in bulk coordinates are given as *hkl* values; those for the CTRs in surface coordinates, as (*h*,*k*). The straight lines along *Q*_*z*_ highlight the positions of the CTRs; the circular lines indicate constant |*Q*| values. For the oxide, the positions of the straight line and powder ring are the result of fitting the individual contributions, as explained in the main text. (b) In-plane peak profiles of the oxide signals at *Q*_*z*_ = 0.075 Å^–1^. For different anodic polarization times two superposed pseudo-Voigt peaks are fitted. As explained in more detail in the main text, the α-PtO_2_ peak, which is present even in the absence of anodic polarization, is shown in gray; the contribution of powderlike β-PtO_2_ is shown in yellow. (c) Enlarged areas around the surface rods of the platinum oxide. Before anodic polarization (0 min), the streak is nearly parallel to the surface normal. After 5 min of anodic polarization at +180 mV, a new diffraction feature appears as explained in the main text.

Although the overlapping of the diffraction signals hampers a detailed extraction of the individual signals for further structural analysis, it is clear that the α-PtO_2_ rod does not show any osillations along *Q*_*z*_. This indicates that the structure is of a 2D nature with a thickness of the order of a single α-PtO_2_ trilayer. The powderlike signal, attributed to the β-PtO_2_ phase, is seen to grow with increasing anodic polarization times. Figure 4c shows the in-plane intensity distribution along *Q*_*r*_ at *Q*_*z*_ = 0.075 Å^–1^ and how the β-PtO_2_ component is initially practically absent, then increases drastically after the first 5 min anodic polarization at +180 mV, and keeps increasing for longer polarization times but at a lower rate. The fact that the β-PtO_2_ phase forms during anodic polarization can only be explained if it appears at the buried interface, because at the free Pt surface there is no driving force for a polarization-driven oxidation and the thin α-PtO_2_ remains unaltered irrespective of the polarization. This means that at the buried interface two different oxide species form: the ultrathin oxide and powderlike β-PtO_2_, the former reversibly and the latter irreversibly with polarization. The slow continuous formation of β-PtO_2_ correlates with the local delamination process, which leads to drastic surface morphological changes in the form of bubbles and/or pyramidlike structures, as observed previously.^6,36^Figure 5 shows SEM and AFM images of the surface before and after the EC experiments described above. These show that around 20% of the surface area is covered by pyramidlike structures and in between smaller roundish blisters have formed. We rule out that these morphological changes are merely thermally induced. First of all, the used temperature is about 350 K lower than the deposition temperature. Furthermore, typical conditions needed for extensive mass transport of Pt on oxides are temperatures above 1300 K for extensive times of 24 h.^37^ Once a thick, closed, and relaxed Pt film has formed at deposition temperature, it will remain morphologically stable at the used experimental temperatures. During anodic polarization, oxygen gas can form at the Pt/YSZ interface and the pressure can increase up to several tens of bars, perhaps even 10–100 times higher during the initial platinum detachment, inside the blisters.^38^ This process, which also must lead to considerable strain in the Pt, has been discussed to result in the lowering of kinetic barriers for oxide formation leading to unusually thick platinum oxide on the inside of the delaminated areas.^6^ At standard pressures, only about 1 monolayer (ML) of platinum oxidizes.^3,39^ From the width of the β-PtO_2_ diffraction peak, see Figure 4c, it is estimated that the 3D domain/grain size is of the order of 10 nm, indeed much more than that observed at standard pressures and also much thicker than the α-PtO_2_ observed here for the outermost free Pt surface. Combined with the results obtained here, it seems likely that this thicker polycrystalline oxide consists of β-PtO_2_, which should also form at slightly higher temperature and/or pressure than α-PtO_2_.^32^ Due to the platinum locally deforming from the initial flat surface, the impinging X-rays locally do not make a grazing angle anymore and thus can reach the inside of the bubbles and pyramids, resulting in the detection of the diffraction signals from these buried areas. The formation of the blisters also results in a reduction of the flat area covered by the α-PtO_2_ and which is seen as a decrease in diffracted intensity (see Figure 4c). The fact that only part of the whole Debye–Scherrer ring is clearly observed may point to heavily preferentially oriented crystallites (see also the Supporting Information). It is conceivable that β-PtO_2_ grows quasi-epitaxially on the inside of the faceted piramidlike structures, as shown in Figure 5.

**Figure 5:**
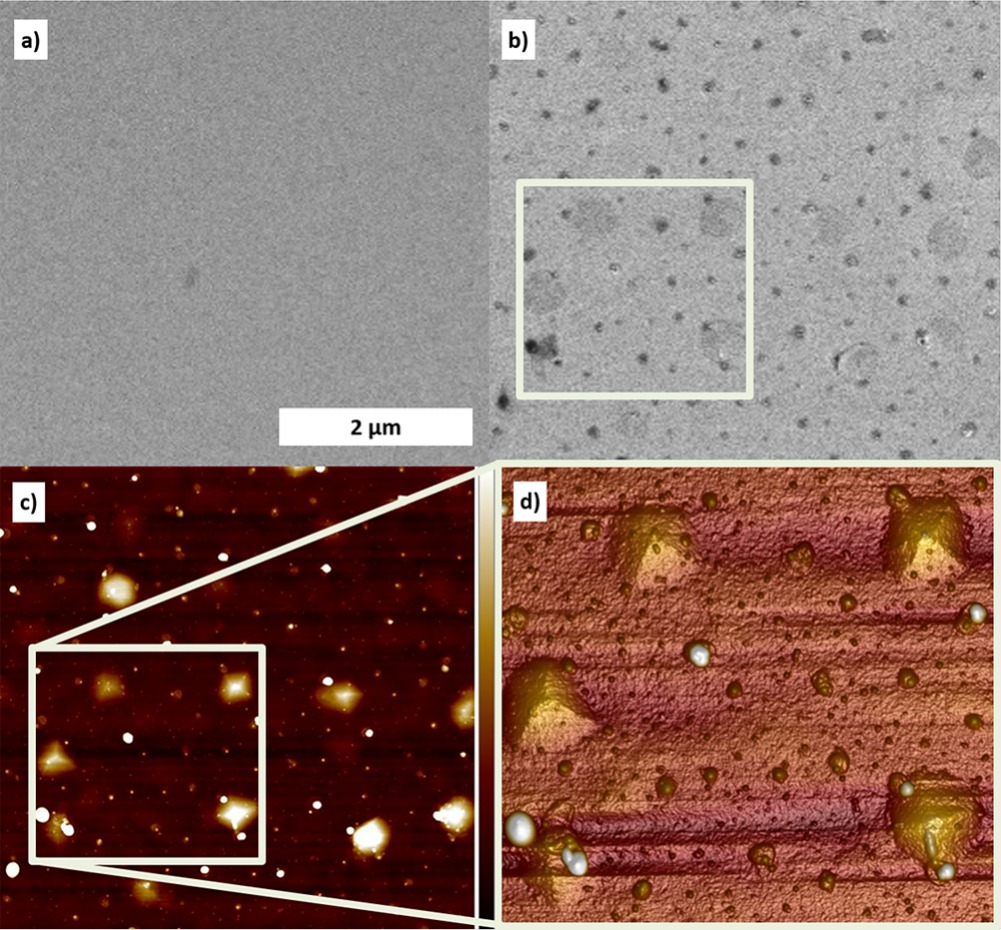
Secondary electron SEM image of the epitaxial Pt film (a) before and (b) after the experiment; (c) corresponding 2D topographic AFM image. The color bar on the right indicates a total height difference of 25 nm. (d) Magnified 3D view of the region indicated by the white square in panels b and c. The scale bar in panel a applies to panels a–c.

Previous spectroscopic results point to an average composition of Pt_3_O_4_ on the inside of the delaminated areas.^6^ Combined with the results obtained here, this could mean that several oxide species have formed and that β-PtO_2_ is the most crystalline and thus detectable by XRD. Such a reasoning is based on the assumption that any platinum oxide belongs to one of the relatively well-known stoichiometric phases. However, nonstoichiometric phases, with Pt in oxidation states other than +2 and +4, have been reported in literature.^40,41^ In addition, the strongest oxide peaks, discussed here to belong to a single trilayer of α-PtO_2_ on the outer electrode surface, would also form the strongest Fourier components of the so-called spoked-wheel reconstruction.^42^ The fact that no superstructure diffraction pattern belonging to such a large unit cell is observed (see Figure 3) leads to the conclusion that either it does not form under the conditions used here or that it forms a minority phase. Similarly, the exact phase and in-plane structure of the ultrathin layer forming reversibly at the buried interface remains elusive.

In conclusion, during electrochemical cycling between the anodic and cathodic regime, an ultrathin oxide forms and dissolves, respectively, at the deeply buried interface between a platinum electrode and a YSZ solid-state electrolyte. This process is observed by directly probing the interface by hard X-ray reflectometry and correlating the results with CV measurements, taken simultaneously. The exact phase of this oxide cannot be determined, because the method delivers the electron density ρ = 2.8*e*/Å^3^, which is compatible with several platinum oxide phases. Under the experimental conditions—a temperature of 702 K and ambient air—the outermost electrode surface also oxidizes. From the measurements of depth-dependent diffraction patterns, it is concluded that a single trilayer of α-PtO_2_ covers the Pt surface. This oxide and its thickness appear completely stable and unaltered upon polarization and resemble the case of thermal oxidation of Pt. Its stability suggests oxygen diffusion from/to the triple phase boundary along the free Pt surface, thus keeping the Pt surface unaffected by electrochemical polarization. After prolonged anodic polarization, the irreversible formation of another oxide phase is observed. Its diffraction peak position suggests that it matches best with polycrystalline preferentially oriented β-PtO_2_. Since the structural evolution of this oxide phase correlates with the anodic polarization time and with a slow local delamination process of the electrode from the YSZ, it is concluded that it forms on the inside of the growing blisters. The formation of β-PtO_2_ inside the blisters is also compatible with the local elevated oxide pressure and contrasts the thermodynamic conditions at the free surface where α-PtO_2_, which can form at standard pressures, is observed. Moreover, the local conditions near and inside the blisters lead to additional strain in the platinum, which may help overcome kinetic barriers for oxidation and thus promote the formation of β-PtO_2_.

## Supporting Information Available

Cyclic voltammetry, in-plane RSMs at different grazing incidence angles, diffraction peak fitting details, extended RSM showing large portion of reciprocal space in the (*Q*_*r*_, *Q*_*z*_)-plane, and XRD measurements taken in the lab during growth optimization (PDF)

## References

[ref1] Zhang S.; Yuan X.-Z.; Hin J. N. C.; Wang H.; Friedrich K. A.; Schulze M. A Review of Platinum-Based Catalyst Layer Degradation in Proton Exchange Membrane Fuel Cells. J. Power Sources 2009, 194, 588–600. 10.1016/j.jpowsour.2009.06.073.

[ref2] Huang Y.-F.; Koper M. T. M. Electrochemical Stripping of Atomic Oxygen on Single-Crystalline Platinum: Bridging Gas-Phase and Electrochemical Oxidation. J. Phys. Chem. Lett. 2017, 8, 1152–1156. 10.1021/acs.jpclett.7b00136. 28225278 PMC5357804

[ref3] Ellinger C.; Stierle A.; Robinson I. K.; Nefedov A.; Dosch H. Atmospheric Pressure Oxidation of Pt(111). J. Phys.: Condens. Mater. 2008, 20, 184013 10.1088/0953-8984/20/18/184013.

[ref4] Miller D. J.; Oberg H.; Kaya S.; Sanchez Casalongue H.; Friebel D.; Anniyev T.; Ogasawara H.; Bluhm H.; Pettersson L. G. M.; Nilsson A. Oxidation of Pt(111) under Near-Ambient Conditions. Phys. Rev. Lett. 2011, 107, 195502 10.1103/PhysRevLett.107.195502. 22181624

[ref5] Jacobse L.; Huang Y.-F.; Koper M. T. M.; Rost M. J. Correlation of Surface Site Formation to Nanoisland Growth in the Electrochemical Roughening of Pt(111). Nat. Mater. 2018, 17, 277–282. 10.1038/s41563-017-0015-z. 29434306

[ref6] Keller T. F.; Volkov S.; Navickas E.; Kulkarni S.; Vonk V.; Fleig J.; Stierle A. Nano-Scale Oxide Formation Inside Electrochemically-Formed Pt Blisters at a Solid Electrolyte Interface. Solid State Ion. 2019, 330, 17–23. 10.1016/j.ssi.2018.11.009.

[ref7] Hejral U.; Vlad A.; Nolte P.; Stierle A. In Situ Oxidation Study of Pt Nanoparticles on MgO(001). J. Phys. Chem. C 2013, 117, 19955–19966. 10.1021/jp404698k.

[ref8] Fantauzzi D.; Krick Calderon S.; Mueller J. E.; Grabau M.; Papp C.; Steinruck H.-P.; Senftle T. P.; van Duin A. C. T.; Jacob T. Growth of Stable Surface Oxides on Pt(111) at Near-Ambient Pressures. Angew. Chem. 2017, 56, 2594–2598. 10.1002/anie.201609317. 28120368

[ref9] Van Spronsen M. A.; Frenken J. W. M.; Groot I. M. N. Observing the Oxidation of Platinum. Nat. Commun. 2017, 8, 429 10.1038/s41467-017-00643-z. 28874734 PMC5585323

[ref10] Drnec J.; Ruge M.; Reikowski F.; Rahn B.; Carla F.; Felici R.; Stettner J.; Magnussen O. M.; Harrington D. A. Initial stages of Pt(111) Electrooxidation: Dynamic and Structural studies by surface X-ray diffraction. Electrochim. Acta 2017, 224, 220–227. 10.1016/j.electacta.2016.12.028.

[ref11] Lanyon M.; Trapnell B. The Interaction of Oxygen with Clean Metal Surfaces. Proc. R. Soc. London 1955, 227, 387–399.

[ref12] Reddy A.; Genshaw M.; Bockris J. Ellipsometric Study Of Oxygen-Containing Films On Platinum Anodes. Journal Of Chemical Physics 1968, 48, 671–675. 10.1063/1.1668699.

[ref13] Jacobse L.; Vonk V.; McCrum I. T.; Seitz C.; Koper M. T.M.; Rost M. J.; Stierle A. Electrochemical Oxidation of Pt(111) Beyond the Place-Exchange Model. Electrochim. Acta 2022, 407, 139881 10.1016/j.electacta.2022.139881.

[ref14] Jaccoud A.; Foti G.; Comninellis C. Electrochemical Investigation of Platinum Electrode in Solid Electrolyte Cell. Electrochim. Acta 2006, 51, 1264–1273. 10.1016/j.electacta.2005.06.026.

[ref15] Pöpke H.; Mutoro E.; Raiss C.; Luerssen B.; Amati M.; Abyaneh M. K.; Gregoratti L.; Janek J. The Role of Platinum Oxide in the Electrode System Pt(O-2)/Yttria-Stabilized Zirconia. Electrochim. Acta 2011, 56, 10668–10675. 10.1016/j.electacta.2011.04.057.

[ref16] Opitz A. K.; Hoerlein M. P.; Huber T.; Fleig J. Current-Voltage Characteristics of Platinum Model Electrodes on Yttria-Stabilized Zirconia. J. Electrochem. Soc. 2012, 159, B502–B513. 10.1149/2.044205jes.

[ref17] Pöpke H.; Mutoro E.; Luerssen B.; Janek J. Oxidation of Platinum in the Epitaxial Model System Pt(111)/YSZ(111): Quantitative Analysis of an Electrochemically Driven PtOx Formation. J. Phys. Chem. C 2012, 116, 1912–1920. 10.1021/jp209645t.

[ref18] Beck G; Fischer H; Mutoro E; Srot V; Petrikowski K; Tchernychova E; Wuttig M; Ruhle M; Luersen B; Janek J Epitaxial Pt(111) Thin Film Electrodes on YSZ(111) and YSZ(100) âĂŤ Preparation and Characterisation. Solid State Ionics 2007, 178, 327–337. 10.1016/j.ssi.2007.01.025.

[ref19] Opitz A. K.; Fleig J. Investigation of O2 Reduction on Pt/YSZ by means of Thin Film Microelectrodes: The Geometry Dependence of the Electrode Impedance. Solid State Ion. 2010, 181, 684–693. 10.1016/j.ssi.2010.03.017.

[ref20] Opitz A. K.; Lutz A.; Kubicek M.; Kubel F.; Hutter H.; Fleig J. Investigation of the Oxygen Exchange Mechanism on Pt|Yttria Stabilized Zirconia at Intermediate Temperatures: Surface Path versus Bulk Path. Electrochim. Acta 2011, 56, 9727–9740. 10.1016/j.electacta.2011.07.112. 22210951 PMC3209560

[ref21] Hutterer A. The Multi-Faceted Aspects of Oxygen Reduction on Platinum Model Electrodes on Yttria-Stabilised Zirconia: Reaction Mechanism, Enhancement and Degradation of Kinetics, Oxygen Storage. Ph.D. thesis, Technical University of Vienna, Vienna, Austria, 2022.

[ref22] Seeck O. H.; Deiter C.; Pflaum K.; Bertam F.; Beerlink A.; Franz H.; Horbach J.; Schulte-Schrepping H.; Murphy B. M.; Greve M.; et al. The High-Resolution Diffraction Beamline P08 at PETRA III. J. Synchrotron Radiat. 2012, 19, 30–38. 10.1107/S0909049511047236. 22186641

[ref23] Bjorck M.; Andersson G. GenX: An Extensible X-Ray Reflectivity Refinement Program Utilizing Differential Evolution. J. Appl. Crystallogr. 2007, 40, 1174–1178. 10.1107/S0021889807045086.

[ref24] Parratt L. Surface Studies of Solids by Total Reflection of X-Rays. Phys. Rev. 1954, 95, 359–369. 10.1103/PhysRev.95.359.

[ref25] Siegel S.; Hoekstra H.; Tani B. Crystal Structure of Beta-Platinum Dioxide. J. Inorg. Nucl. Chem. 1969, 31, 3803–3807. 10.1016/0022-1902(69)80300-3.

[ref26] Hoekstra H.; Siegel S.; Gallagher F. Reaction of Platinum Dioxide with some Metal Oxides. Adv. Chem. Ser. 1971, 98, 39–53. 10.1021/ba-1971-0098.ch004.

[ref27] Feidenhans'l R. Surface-Structure Determination by X-Ray-Diffraction. Surf. Sci. Rep. 1989, 10, 105–188. 10.1016/0167-5729(89)90002-2.

[ref28] Moore W.; Pauling L. The Crystal Structures of the Tetragonal Monoxides of Lead, Tin, Palladium, and Platinum. J. Am. Chem. Soc. 1941, 63, 1392–1394. 10.1021/ja01850a074.

[ref29] Kumar J.; Saxena R. Formation of Na Cl- and Cu2 O-type Oxides of Platinum and Palladium of Carbon and Alumina Support Films. J. Less-Comm. Met. 1989, 147, 59–71. 10.1016/0022-5088(89)90148-3.

[ref30] McBride J.; Graham G.; Peters C.; Weber W. Growth and Characterization of Reactively Sputtered Thin-Film Platinum Oxides. J. Appl. Phys. 1991, 69, 1596–1604. 10.1063/1.347255.

[ref31] Range K.; Rau F.; Klement U.; Heyns A. Beta-Pt O2: High Pressure Synthesis of Single Crystals and Structure Refinement. Mater. Res. Bul. 1987, 22, 1541–1547. 10.1016/0025-5408(87)90220-0.

[ref32] Muller O.; Roy R. Formation and Stability of the Platinum and Rhodium Oxides at High Oxygen Pressures and the Structures of Pt3O4, beta-PtO2 and RhO2. J. Less-Comm. Met. 1968, 16, 129–146. 10.1016/0022-5088(68)90070-2.

[ref33] Busch R.; Cairo A.; E.E G.; J R. Preparacion y Estructura Cristallina del PtO2. Rev. Union Mater. Argentina 1951, 15, 16–17.

[ref34] Herreor Fernandez M. P.; Chamberland B. A New High Pressure Form of PtO2. J. Less-Common Met. 1984, 99, 99–105. 10.1016/0022-5088(84)90338-2.

[ref35] Galloni E.; Roffo A. The Crystalline Structure of Pt3O4. J. Chem. Phys. 1941, 9, 875–877. 10.1063/1.1750860.

[ref36] Pöpke H.; Mutoro E.; Luerssen B.; Janek J. The Potential of In-situ Scanning Electron Microscopy - Morphology Changes of Electrically Polarized Thin Film Pt(O2)/YSZ Model Electrodes. Solid State Ion. 2011, 189, 56–62. 10.1016/j.ssi.2011.02.021.

[ref37] Dupraz M.; Li N.; Carnis J.; Wu L.; Labat S.; Chatelier C.; van de Poll R.; Hofmann J. P.; Almog E.; Leake S. J. Imaging the Facet Surface Strain State of Supported Multi-faceted Pt Nanoparticles during Reaction. Nat. Commun. 2022, 13, 3003 10.1038/s41467-022-30592-1. 35637233 PMC9151645

[ref38] Mutoro E.; Günther S.; Luerssen B.; Valov I.; Janek J. Electrode Activation and Degradation: Morphology Changes of Platinum Electrodes on YSZ during Electrochemical Polarisation. Solid State Ionics 2008, 179, 1835–1848. 10.1016/j.ssi.2008.05.007.

[ref39] Ackermann M.; Pedersen T.; Hendriksen B.; Robach O.; Bobaru S.; Popa I.; Quiros C.; Kim H.; Hammer B.; Ferrer S. Structure and Reactivity of Surface Oxides on Pt(110) During Catalytic CO Oxidation. Phys. Rev. Lett. 2005, 95, 255505 10.1103/PhysRevLett.95.255505. 16384470

[ref40] Mom R.; Frevel L.; Velasco-Velez J.-J.; Plodinec M.; Knop-Gericke A.; Schloegl R. The Oxidation of Platinum under Wet Conditions Observed by Electrochemical X-ray Photoelectron Spectroscopy. J. Am. Chem. Soc. 2019, 141, 6537–6544. 10.1021/jacs.8b12284. 30929429 PMC6727372

[ref41] Grande B.; Müller-Buschbaum H. Ein Beitrag zu Verbindungen vom Typ MexPt3O4. J. Inorg. Nucl. Chem. 1977, 39, 1084–1085. 10.1016/0022-1902(77)80270-4.

[ref42] van Spronsen M. A.; Frenken J. W. M.; Groot I. M. Observing the oxidation of platinum. Nat. Commun. 2017, 8, 429 10.1038/s41467-017-00643-z. 28874734 PMC5585323

