# Supplementary material for: Reversible Ultrathin PtOx Formation at the Buried Pt/YSZ(111) Interface Studied In Situ under Electrochemical Polarization
Source: J Phys Chem Lett. 2023 Feb 17;14(8):2065–71. doi: 10.1021/acs.jpclett.2c03614 (PMC9986955; doi:10.1021/acs.jpclett.2c03614)
Supplement: Supplementary file 1 — jz2c03614_si_001.pdf [file jz2c03614_si_001.pdf]

Supporting information for: Reversible Ultrathin PtO_x Formation at the Buried Pt/YSZ(111) Interface studied In situ under Electrochemical Polarization

Vedran Vonk,[*,†] Sergey Volkov,[‡] Thomas F. Keller,[†,§] Alexander Hutterer,[¶] Pirmin Lakner,[†] Florian Bertram,[‡] Jürgen Fleig,[¶] Alexander K. Opitz,[*,¶] and Andreas Stierle[†,§]

†*Centre for X-ray and Nanoscience CXNS, Deutsches Elektronen-Synchrotron DESY, Notkestr. 85, 22607 Hamburg, Germany*

‡*Deutsches Elektronen-Synchrotron DESY, Notkestr. 85, 22607 Hamburg, Germany*

¶*TU Wien, Institute of Chemical Technologies and Analytics, 1060 Vienna, Austria*

§*University of Hamburg, Physics Department, 20355 Hamburg, Germany*

E-mail: vedran.vonk@desy.de; alexander.opitz@tuwien.ac.at

In the folowing, additional information is given concerning the electrochemical conditions during the experiments and more details concerning the x-ray data analysis.

Cyclic Voltammetry

Electrochemical impedance spectroscopy (EIS) and cyclic voltametry (CV) measurements were performed to confirm the correct functioning of the cell and to identify the cathodic

and anodic peaks, which are a fingerprint for the formation and dissolution of platinum oxide species.. The sample cell was heated to an operating temperature of 702 K. X-ray measuerements before and after potential sweeps from the cathodic (-700 mV) to the anodic (+180 mV) regime were performed. In addition, staying at the conditions, where, according to electrochemical studies, PtOx should be present at the interface, we performed the same set of measurements as with non-biased sample. Figure S1 shows some of the CV data and corresponding reflected x-ray intensities while cycling the voltage at 5 mV/s. Detailed XRR measurements, taken in-situ, while keeping the sample at cathodic or anodic polarizations are shown in the main text.

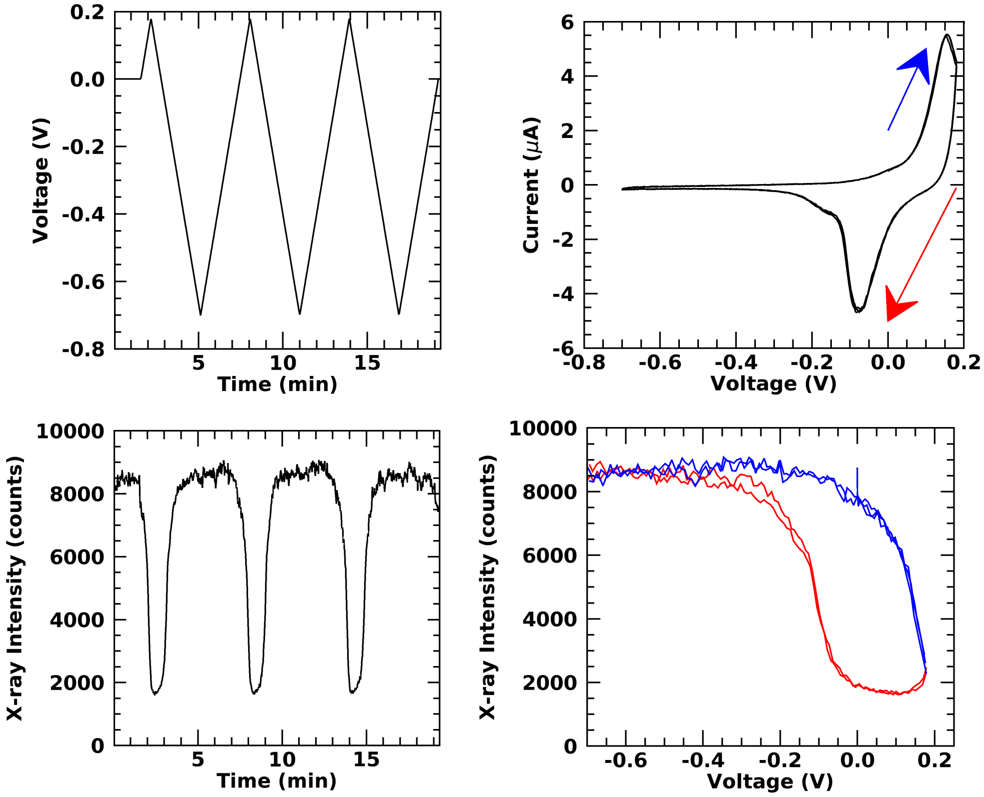

Figure S1: CV curves measured during the operando x-ray measurements and corresponing reflected intensity. (top left) Voltage vs. time. (top right) CV measurements during 3 cycles. The arrows indicate the positive voltage increase direction (blue) and negative (red). (bottom left) Reflected intensity at a Q=0.61 Å^{-1} and (bottom right) reflected intensity vs polarization.

Grazing-incidence XRD

Due to refraction and absorption effects, an x-ray beam has a finite penetration depth when using a so-called extended face crystal with very low roughness and Bragg diffraction geometry. In particular in grazing incidence-exit geometry near the angle for total external reflection, the penetration depth can become very small, of the order of several nm. The crystal volume from which diffraction is observed is mostly determined by the so-called information depth Λ, which is given by:

$$\frac{1}{\Lambda} = \frac{1}{\Lambda_i} + \frac{1}{\Lambda_f} \tag{1}$$

with the subscripts i and f indicating the incoming and outgoing beams, respectively. The individual contributions are:

$$\frac{1}{\Lambda_{i,f}} = \frac{2\pi}{\lambda} \left[(2\delta - \sin^2 \alpha_{i,f}) - \left((\sin^2 \alpha_{i,f} - 2\delta)^2 + 4\beta^2 \right)^{\frac{1}{2}} \right]^{\frac{1}{2}} \tag{2}$$

with λ the x-ray wavelength, δ the real and β the imaginary part of the refractive index and $\alpha_{i,f}$ the angle of the incoming and outgoing beam with respect to the surface plane. Figure S2 shows the in-plane reciprocal space maps take with 2 different angles of incidence $\alpha_i = 0.2°$ and $2.0°$. The outgoing angles for these measurements were approx. $\alpha_f = 0.8°$ and $1.2°$, respectively, which are several times the critical angle for total external reflection $\alpha_c = 0.2°$ and therefore do not limit the information depth ($\Lambda_f >> \Lambda_i \rightarrow \Lambda \simeq \Lambda_i$).

Peak Positions Platinum Oxide

The x-ray measurements whereby only the outermost surface contributed to the diffraction patterns shows two partially overlapping platinum oxide peaks, as also discussed in relation to Fig. 4 in the main text. Figure S3 shows the results of fitted peak positions in Q_r for different Q_z along the diffraction feature.

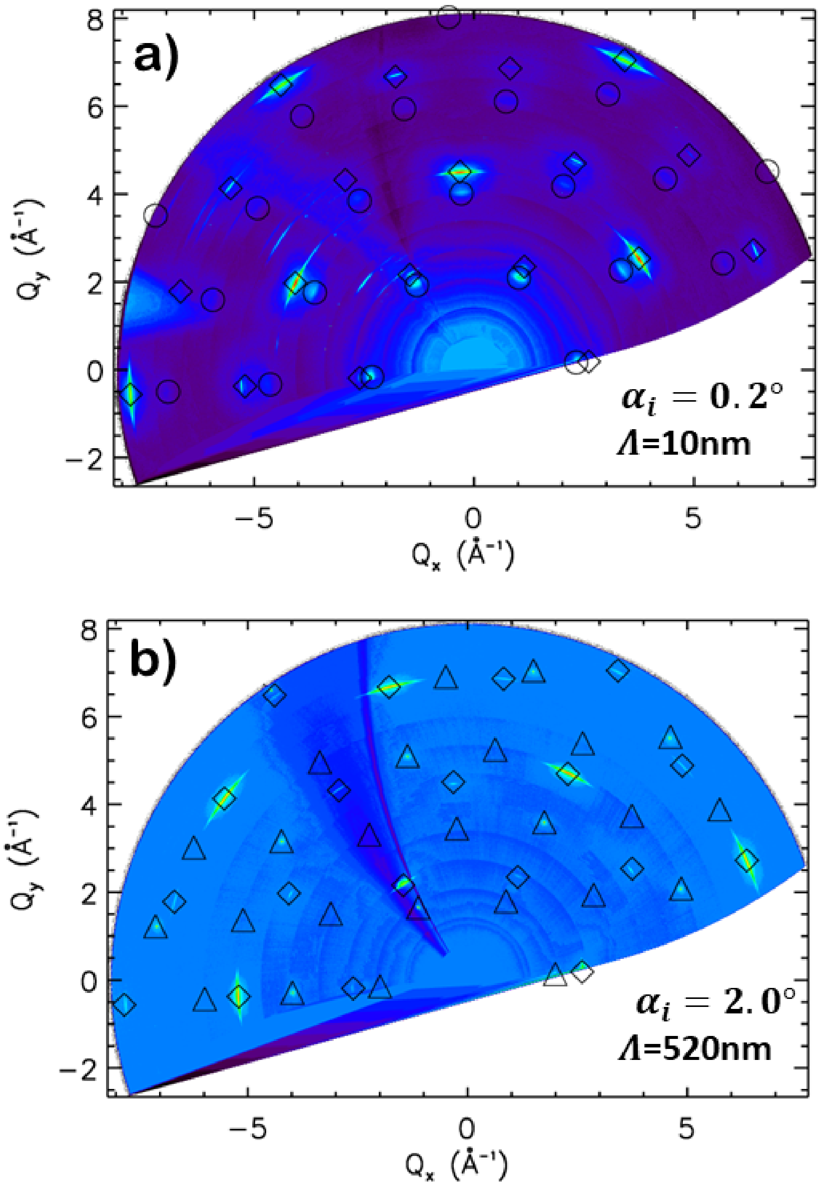

Figure S2: Reciprocal space maps taken at 702 K and U=-500 mV polarization. a) At an incidence angle $\alpha_i = 0.2$ deg, the x-ray beam penetrates only $\Lambda = 10$ nm into the Pt electrode, which leads to the observation of CTR signal from the Pt metal (diamonds) and surface platinum oxide (circles). b) At an incidence angle $\alpha_i = 2.0$ deg, the x-ray beam would have a penetration depth $\Lambda = 520$ nm into Pt and therefore goes completely through the electrode and reaches the interface with the YSZ substrate. The transmission through the Pt is also high enough that any scattering signal can come back. This leads to the observation of CTR signal from the Pt metal (diamonds) and YSZ (triangles). Due to the higher background signal as compared with a) the surface oxide peaks are not clearly visible and their positions are omitted for clarity.

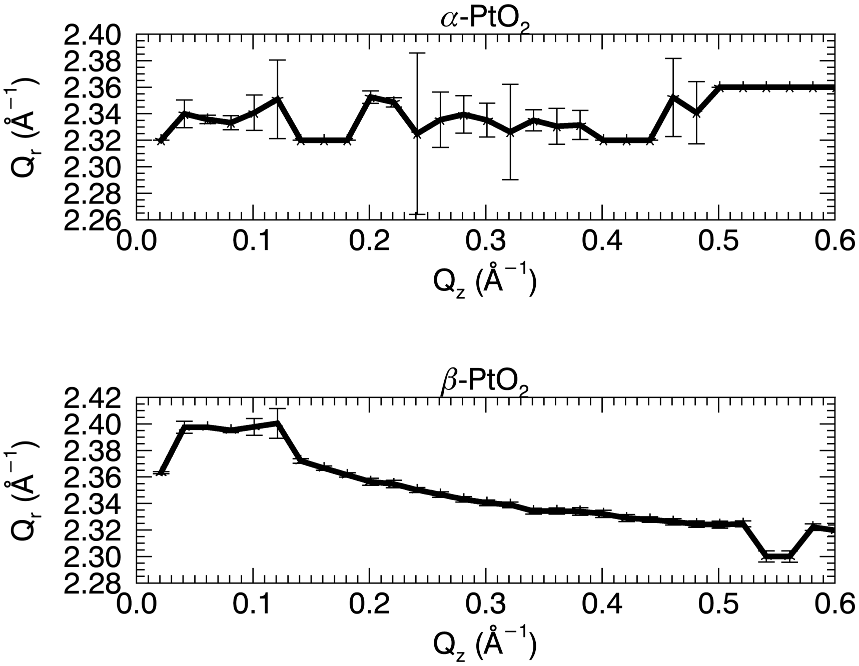

Figure S3: Results of fitting the superposition of two Pseudo-Voigt peaks to the line profiles along Q_r at different Q_z positions. (top) In-plane (Q_r) peak position from the 2D α-PtO_2 oxide is constant along Q_z. (bottom) The in-plane (Q_r) positions of the peak originating from β-PtO_2 is not constant.

Clearly, the peak position of the α-PtO_2 appears at constant $Q_r = 2.34\text{Å}^{-1}$, which is indicative of a rodlike signal arising from a 2D crystalline surface structure. The peak belonging to β-PtO_2 gradually shifts to lower Q_r values for increasing Q_z. The resulting total momentum transfer $Q = \sqrt{Q_r^2 + Q_z^2}$ is found to be nearly (within the error bar) constant, which is indicative of a powderlike diffraction ring originating from polycrystalline material.

Figure S4 shows an RSM combining the planes which include the (1,0) and (0,1) CTRs. Use is made of the Friedel pairs, which means that any hkl is equivalent with $\bar{h}\bar{k}\bar{l}$. Here this means that $(0, 1, L)$ is equivalent with $(0, \bar{1}, \bar{L})$, which in turn is symmetry equivalent with $(1, 0, \bar{L})$.

The tilt directions of the α-PtO_2 rods at positive and negative L values are not the same. This rules out that it is due to a misorientation (like a small angle grain boundary) of the oxide, but is compatible with a diffraction ring as discussed in relation to Fig. S3. Please note that the miscut of the YSZ substrate is negligibly small and that the Pt metal film grows completely parallel to the surface normal, as can be seen by the direction of the Pt CTR. There is a peak visible around $(Q_r, Q_z) = (2, -1.4)$, which has the same $|Q|$-value as the β-PtO_2 peaks found to overlap with the α-PtO_2 rod. Although it is not completely clear at the moment, this would be compatible with the preferential orientation of the β-PtO_2 as dictated by some of the strongly facetted delaminated areas.

Film growth optimization

During the optimization of the film growth, XRD measurements have routinely been carried out in the lab. Typical measurements from which it is concluded that the film is epitaxial are shown in fig. S5.

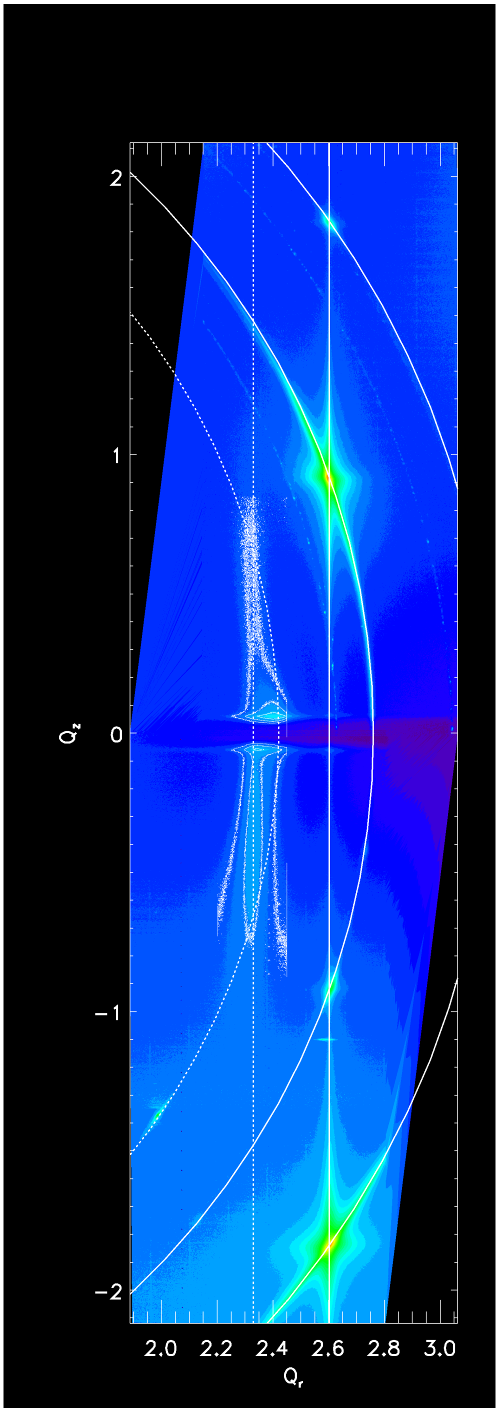

Figure S4: RSM in the (Q_r, Q_z)-plane including the (1,0) and (0,1) Pt CTRs, whereby the latter is plotted as $(1, 0, -L)$ (Friedel rod). The signal from platinum oxide, which is here highlighted by additional contours, shows to be tilted. The dashed straight line and circle indicate the individual oxides (see Fig. S3 and the full circles indicate the constant Q values for two different metal Pt peaks.

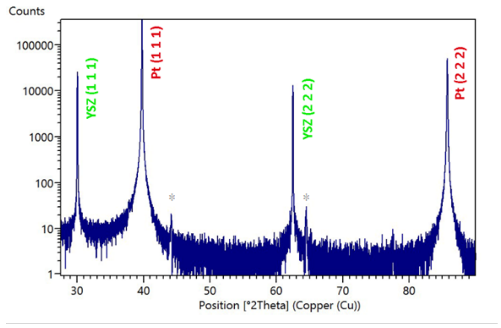

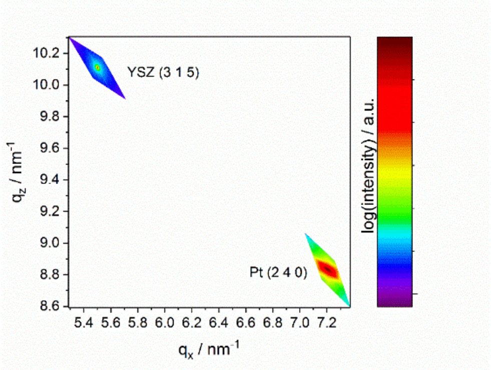

Figure S5: (left) θ-2θ XRD (the asterisks indicate signals from the sample holder) and (right) RSM measurements using a Cu K$_\alpha$ lab source of a Pt film grown around 1050 K.
